# Greens in the Gaps: Diversity and the Ecological Potential of Urban Spontaneous Vegetation in Sidewalk Ecosystems

**DOI:** 10.3390/plants14162542

**Published:** 2025-08-15

**Authors:** Nadchawan Charoenlertthanakit, Angkhana Inta, Dia Panitnard Shannon, Boonchuang Boonsuk, Pimonrat Tiansawat

**Affiliations:** 1 Department of Biology, Faculty of Science, Chiang Mai University, Chiang Mai 50200, Thailand; nadchawan_c@cmu.ac.th (N.C.); angkhana.inta@cmu.ac.th (A.I.); dia.shannon@cmu.ac.th (D.P.S.); 2 Forest Restoration Research Unit, Faculty of Science, Chiang Mai University, Chiang Mai 50200, Thailand; 3 Plant Biology Research Unit, Program in Biology, School of Science, University of Phayao, Phayao 56000, Thailand; boonchuang.bo@up.ac.th

**Keywords:** sustainable urban greening, sidewalk vegetation, tropical cities, medicinal plants, non-native species, pavement flora, urban biodiversity

## Abstract

Green spaces are essential for urban environments, but urban expansion often results in fragmented patches and narrow pavements unsuitable for tree growth. Consequently, there is a pressing need for alternative vegetation in urban landscapes where tree planting is impractical. Urban spontaneous vegetation (USV)—plants that establish naturally without cultivation—shows promise for urban landscaping, and yet has been underexplored in urban ecology. This study was the first systematic survey to examine the composition of USV in Chiang Mai, Thailand, across seven urban locations. The survey was conducted along 13 sidewalk routes (totaling 33.24 km), documenting all non-tree vascular plant species. A total of 63 USV species from 24 families were recorded, predominantly colonizing pavement gaps, cracks, and curbside cracks. The most diverse family was Poaceae, with 15 species. Among the 61 identified species, 32 species (52%) were non-native. Seven species were found in all surveyed locations, highlighting their adaptability to challenging urban conditions. Fifty USV species are medicinal plants. Many species exhibit characteristics that are ideal for sustainable landscaping, such as drought tolerance, low maintenance requirements, and ornamental value. This study highlights USV as a key component of green infrastructure and provides new insights for urban sustainable landscaping.

## 1. Introduction

Preserving biodiversity in cities enhances resilience against climate impacts and supports long-term sustainability goals [1,2]. Increasing urban green spaces is essential for sustainable urban systems, as vegetation provides ecosystem services such as air purification, cooling, stormwater regulation, and recreational opportunities [3,4,5,6]. Strategies such as planting trees and creating public parks are widely recognized as effective nature-based solutions for expanding urban green space [7,8]. However, as urban areas develop, the growing number of roads and buildings leave fragmented patches and/or narrow pavements unsuitable for growing trees [9]. Thus, the identification of alternative vegetation for urban landscaping is necessary to sustain urban biodiversity, particularly in areas where trees cannot be planted.

Vegetation in urban environments falls into three broad categories: (1) planted vegetation in managed areas, including parks and street trees; (2) remnant natural habitats; and (3) spontaneous vegetation [10]. Urban spontaneous vegetation (USV) refers to plants that establish naturally without cultivation [11] and are not remnants of natural habitats [10]. USV plays a pivotal role in ecological regeneration within urban areas and provides a variety of ecosystem services [11]. Some USV impose minimal costs on managers [12,13,14]. Urban spontaneous plants can thrive in harsh substrates, such as hard surfaces that lack rooting space, and have limited substrate areas, low moisture availability, and experience disturbances from trampling or urban pollution [14,15,16,17]. Despite these benefits, unmanaged USV may also pose challenges in pedestrian areas. For example, the growth of certain species can disrupt hard surfaces, create uneven pavements, and potentially hinder pedestrian accessibility or clog storm drains [14,15,16,18,19].

USV studies were started in European countries in the 1970s to find alternatives for low-maintenance green spaces in urban areas. In Europe, USV (e.g., perennials, herbaceous dicotyledonous species, non-graminoid monocotyledonous plants, and geophytes) was used in urban meadows because it provides ecosystem services similar to lawns but requires less maintenance [13]. For other countries, such as China, Japan, Iran, and India, the study of USV has only recently received more attention from ecologists. Their studies focus on the composition, diversity, and response mechanisms of USV to urban conditions. It was found that most of the USV in each study area were herbaceous plants, and most of the families were Poaceae and Asteraceae [10,20,21,22]. In Thailand, most studies on spontaneous vegetation have focused on weed control in agricultural areas [23,24,25,26,27]. There has been no study conducted in urban areas.

This study is the first to provide information about USV in the highly developed city of Chiang Mai, Thailand. It aims to determine the species composition and morphological characteristics of USV on urban sidewalks. By identifying species that naturally thrive in these challenging urban conditions, this study contributes to a broader understanding of low-maintenance, biodiversity-friendly landscaping strategies for tropical cities. The findings provide the list of species that can inform future urban greening initiatives, particularly in drought-prone, space-limited areas unsuitable for tree planting.

## 2. Results

### 2.1. Pavement Type and Species Found

**Figure 1:**
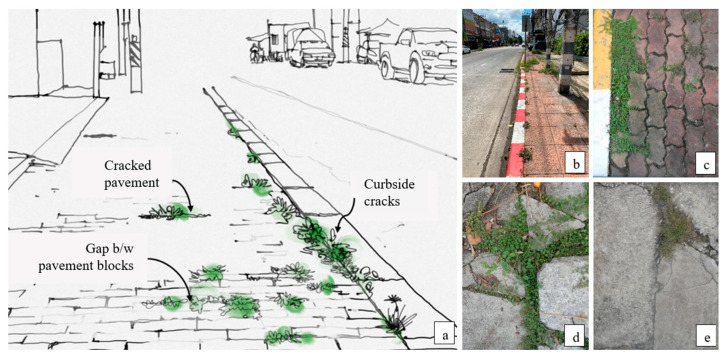
USVs on the Chiang Mai roadside (**a**). USVs predominantly thrived in curbside cracks (**b**), tile pavement gaps (**c**), cracked tile (**d**), and solid (**e**) pavement, along urban roads.

The roadside pavement consists of two main types of surfaces: (1) solid pavement, such as monolithic concrete pavement or stamped concrete; and (2) tile pavement, which comprises various sizes and types of tiles. USV emerges on curbside cracks of the road, gaps between tile pavements, and cracked pavements. Additionally, in solid pavement, USV is commonly found in regions exhibiting pavement deterioration (Figure 1).

**Figure 2:**
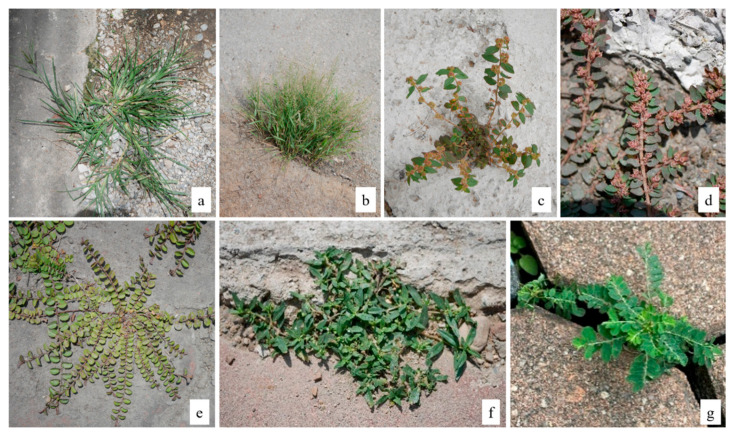
The seven species commonly found in all seven surveyed locations were *Eleusine indica* (**a**), *Eragrostis tenella* (**b**), *Euphorbia hirta* (**c**), *Euphorbia thymifolia* (**d**), *Evolvulus nummularius* (**e**), *Odenlandia corymbosa* (**f**), and *Phyllanthus amarus* (**g**).

In total, there were 61 identified species and 2 unknown species of USV (Table 1). Two species remained unknown due to unsuitable phenological phases for identification in the survey. Moreover, the permanent elimination of USVs by the city’s staff significantly hinders subsequent efforts to identify these two species. The USV found in this study belonged to 24 families and 49 genera. The most represented USV families were Poaceae (15 species) and Asteraceae (8 species). Poaceae and Asteraceae families accounted for 23 species, representing ~37% of the total recorded USV species. There were seven species—*Eleusine indica*, *Eragrostis tenella*, *Euphorbia hirta*, *Euphorbia thymifolia*, *Evolvulus nummularius*, *Odenlandia corymbosa*, and *Phyllanthus amarus*—found across all surveyed locations (Figure 2).

**Figure 3:**
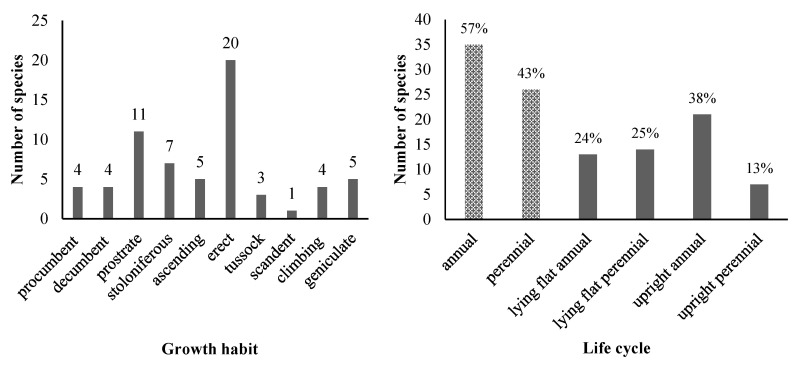
The number of USV species by growth habit (**left**) and life cycle (**right**). In the right-hand side graph, the values on top of the bars show the percentage out of the total of 63 species.

The surveyed USV comprised annual plants (57% of the total species) and perennial plants (43% of the total species) (Figure 3). In terms of plant life cycle, upright annual species had the highest proportion of 38%, followed by lying flat perennial species (25%), lying flat annual species (24%), and upright perennial species (13%) (Figure 3). Among lying flat species, four distinct habits were observed: prostrate (11 species), stoloniferous (7 species), procumbent (4 species), and decumbent (4 species). Upright species encompassed four habits: erect (20 species), ascending (5 species), tussock (3 species), and scandent (1 species). Additionally, geniculate growth habits represented five species.

### 2.2. Native Species of Thailand

Of the 61 species recorded, 29 (48%) were native to Thailand. The most represented family among native species was Poaceae, with 9 species, *Eleusine indica* and *Eragrostis tenella* among them, both of which were recorded at all surveyed locations (Figure 2).

Most native species (16 species) exhibited an annual life cycle. In terms of growth habit, many species were lying flat, such as *Grona triflora* and *Indigofera hendecaphylla*, which are both members of the Fabaceae family (Table 1). *Oldenlandia corymbosa* (Figure 2) was found at all surveyed locations and exhibited two growth habits: erect and procumbent.

### 2.3. Alien and Invasive Alien Species

A total of 32 alien species accounted for 52% of the USV recorded. Most of these alien species (19 species) were annual plants, and the majority exhibited an upright growth habit. Some species displayed two distinct growth habits within the same species; for example, *Euphorbia hirta* appeared in both erect and prostrate forms, while *Portulaca pilosa* showed prostrate and ascending habits. Overall, 53% of the alien species belonged to the family Asteraceae, with *Bidens pilosa* and *Tridax procumbens* being notable members of this group (Table 1).

Among the alien species, 19 (31% of all USV) were identified as invasive alien species in Thailand. Of the seven species recorded at all surveyed locations, four were alien species—including *Euphorbia hirta*, *Evolvulus nummularius*, and *Phyllanthus amarus*—all of which are recognized as invasive (Figure 2).

## 3. Discussion

### 3.1. Urban Roadside Habitats Provide Living Space for USVs of Different Growth Habits and Life Cycles

Urban roadside microhabitats, particularly in pavement gaps and curbside cracks, predominantly provide habitats for USVs. It is likely that wider gaps or cracked pavements facilitate the accumulation of rooting substrates, which provide essential resources for plant growth, such as water and nutrients. Nutrients on urban pavements can originate from multiple sources, including decaying organic matter (e.g., leaves and plant debris), wind-blown dust and soil particles, animal waste, rainwater runoff carrying dissolved nutrients, pollution deposits, and human activities such as littering. These varied nutrient sources contribute to the formation of microhabitats that support both plant growth and the presence of USV [82,83].

Despite spatial limitations, environmental disturbances, and high trampling pressure, roadside habitats can support a diverse range of USVs, with 63 species found across 24 families. These results underscore the ecological potential of constrained urban environments to sustain biodiversity despite harsh conditions. The results align with previous studies that identified Poaceae and Asteraceae as the most common spontaneous plant families in urban areas in China [10,84,85,86] and Japan [20,21,22]. Species from these two families are more prevalent than others among both native and alien species globally [87], and they are widespread, possessing a broad ecological niche that allows them to adapt to diverse habitats [85].

Morphological and physiological adaptations enable Poaceae species to thrive in arid and disturbed urban environments. In this study, grasses such as *Cynodon dactylon*, *Eleusine indica*, and *Dactyloctenium aegyptium* were found growing on compacted soils and roadside pavements. These species are known for drought-tolerance traits. For instance, *C. dactylon* has a deep root system, high water-use efficiency, and the ability to activate antioxidant enzymes and accumulate osmolyte under water stress [88,89]. *E. indica* has thickened epidermal layers and expanded cortical cells, which reduce water loss and give aridity tolerance. *D. aegyptium* adapts by reducing leaf area and closing stomata under water deficit conditions, thereby maintaining growth and water-use efficiency [90]. Other species recorded in the study, such as *Eragrostis tenella* and *Chloris barbata*, have structural traits—enlarged vascular bundles, sclerenchyma reinforcement, and Kranz anatomy—that enhance survival and photosynthetic efficiency in a semi-arid environment [91,92].

In addition to morphological and physiological traits, species reproductive strategies enhance the ability to colonize harsh urban habitats. *E. indica* can produce over 120,000 seeds per plant and establish within 38 days [93]. *C. dactylon* spreads vegetatively through stolons and rhizomes, facilitating regeneration even in challenging conditions [94]. Asteraceae species such as *Tridax procumbens* and *Cyanthillium cinereum* exhibit seed and fruit traits that promote survival and dispersal in disturbed urban microhabitats. Both produce dry, single-seeded achenes topped with pappus structures, enabling effective wind dispersal across compacted and fragmented substrates [95,96]. These reproductive adaptations support their rapid colonization of drought-prone, space-limited, and low-maintenance urban environments.

In our study, almost 60% of USVs were annual plants. When comparing the proportions of growth habit and life cycle, upright annual USVs had the highest proportion. This is consistent with a study conducted in Xi’an, China, which found that most herbaceous species along urban roadsides were annuals with an upright growth habit [10]. Annual plants tend to dominate urban areas due to their adaptability to disturbed environments. Their rapid life cycles allow them to complete growth and reproduction before further disruptions occur. These traits, along with their ability to colonize open spaces and outcompete perennials for limited resources, make annual plants more prevalent in urban settings, particularly in poor, compacted soils [97,98,99,100] and in areas with high disturbance by human management [84].

### 3.2. Functional Roles and Greening Potential of USVs

USVs exhibit a range of functional traits that position them as strong candidates for low-maintenance urban greening. Traits such as drought tolerance, compact growth habits, and rapid reproduction enable USVs to thrive in degraded soils and overlooked microhabitats. These characteristics support their persistence in challenging urban environments and contribute to important ecological functions, including enhancing vegetative cover and providing habitats for various organisms.

**Figure 4:**
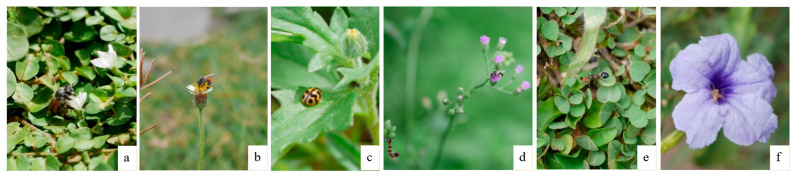
USV supports pollinators and insects: (**a**) A honeybee on the flower of *Evolvulus nummularius*; (**b**) A honeybee landing on the flower of *Tridax procumbens*; (**c**) A ladybird beetle on the leaf of *T. procumbens*; (**d**) A stingless bee and a caterpillar on the flower of *Cyanthillium cinereum*; (**e**) A *Meranoplus bicolor* ant on *Grona triflora*; and (**f**) A lynx spider on the petal of *Ruellia tuberosa*.

Although this study did not formally assess habitat functions, repeated field observations of insects visiting *Evolvulus nummularius* and *Tridax procumbens* suggest that these USVs contribute to pollinator activity in urban areas (Figure 4a–c). Occasional sightings of other arthropods on various USVs (Figure 4d–f) further indicate potential faunal interactions. Similar findings from urban studies have emphasized the role of spontaneous vegetation in boosting pollinator richness within parks and abandoned lots [101,102,103,104,105]. These findings underscore the ecological potential of USVs beyond just vegetative cover.

**Figure 5:**
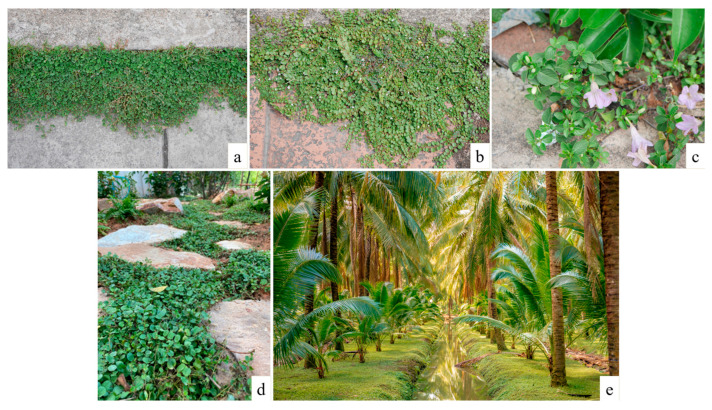
(**a**) *Grona triflora*, (**b**) *Evolvulus nummularius*, and (**c**) *Ruellia prostrata* exhibit a creeping, prostrate growth habit, making them effective ground covers that soften hard edges in the landscape and fill gaps between larger plants or hardscape elements such as pathways and rocks. (**d**) *E. nummularius* used as ground cover in a garden setting, and (**e**) as understory vegetation in a coconut orchard in Thailand.

Many species can be alternatives to conventional turfgrass in urban settings. Low-growing species such as *Grona triflora*, *Evolvulus nummularius*, and *Ruellia prostrata* commonly form dense, mat-like vegetation that spreads horizontally across bare ground (Figure 5a–c). This growth habit aids soil stabilization and visual integration in the landscape. In addition, their minimal maintenance requirements—requiring little to no irrigation or mowing—further enhance their value for sustainable landscape management. Landscape designers in Thailand have recently adopted *E. nummularius* as a turfgrass substitute (Figure 5d) and as understory vegetation in coconut orchards to reduce mowing and herbicide use (Figure 5e). Meanwhile, flowering groundcovers such as *R. prostrata* contribute to ecological aesthetics—a principle that emphasizes the emotional, cultural, and visual appeal of green spaces [106,107]—potentially encouraging public acceptance and appreciation of spontaneous urban flora.

Cultural ecosystem services further enrich the value of USVs. For example, *Cynodon dactylon* (Ya Praek) plays a key role in Thailand’s teacher appreciation ceremony (Pitee Wai Kru) [108], while *Oxalis corniculata* serves traditional purposes such as cleaning silverware [109]. Additionally, over 80% (50 species) of the USVs recorded in this study have been reported in the scholarly literature to exhibit pharmacological properties, including wound healing, anti-inflammatory, antioxidant, and antimicrobial activities (Table 1), highlighting their relevance to local knowledge and practices.

### 3.3. Alien Species in Urban Environments

The ecological roles of alien plants in urban environments are still not well understood. While some alien species may contribute to urban ecosystem services, such as enhancing vegetative cover or supporting pollinators, others may outcompete native species and become invasive, posing ecological risks [110,111]. This study adopts a neutral stance on the presence of alien species in cities. The primary objective of this study was not to assess the status of non-native species or propose pavement management strategies. The study provides information on what USV species are alien and invasive alien species (Table 1).

More than half (52%) of the identified USVs were alien species, aligning with previous research suggesting that urban areas act as hotspots for alien species [112]. Previous studies have shown that alien plant species often outperform native species in terms of growth, reproduction, and resistance to natural enemies [113]. Moreover, as urbanization intensifies, native species numbers tend to decline [114,115,116]. In Asia, the number of recognized alien plant species is already substantial, and their spread is expected to increase further due to ineffective management, changes in land use and climate, and the expansion of international trade, travel, and transportation [117].

Being easy to disperse and escaping natural enemies allows alien species to thrive in urban environments. For example, *Bidens pilosa* and *Tridax procumbens* are globally recognized invasive species, particularly in tropical and subtropical regions [118]. Seeds of *B. pilosa* and *T. procumbens* are wind-dispersed, allowing them to effectively colonize disturbed environments [20,21]. In addition, small seeds of *Euphorbia thymifolia* and *Oldenlandia corymbosa* may be dispersed easily through trampling by pedestrians or by attaching to the wheels of vehicles. Additionally, these species likely benefit from escaping pathogens and herbivores, as well as possessing broad habitat adaptability, allowing them to thrive in urban environments [87]. It is important to recognize the invasive alien species and prevent their spread into natural and/or protected ecosystems, where risks to biodiversity and ecological integrity are significantly higher.

### 3.4. Integrating USVs into Urban Design and Management: Implications and Research Needs

Rather than viewing USVs as nuisances, urban planners and designers can harness their ecological resilience and cultural significance to reduce management costs, promote biodiversity, and reinforce local identity. Future research should aim to identify trait combinations that optimize ecosystem service delivery across diverse urban contexts. However, integrating USVs into urban design must be approached with care. Some species, such as *Cenchrus echinatus*, produce sharp burs that may pose risks to pedestrians and animals. Such examples highlight the need for trait-based evaluations to distinguish beneficial from potentially harmful species.

In addition, urban pavement maintenance should consider both the function of urban spaces and the composition of USV species present. In densely built-up grey spaces, such as metropolitan areas, the spread of USVs is typically limited due to the restrictive nature of existing pavement materials. However, where invasive alien species are detected, more intensive removal efforts are warranted to prevent their further spread. Conversely, in areas where USVs are native or consist of non-invasive alien species, reduced pavement maintenance may be sufficient to support their persistence. Future research should examine how adaptive maintenance regimes—those that permit USVs to persist in stable microhabitats while actively managing invasive species—can help balance biodiversity conservation with urban infrastructure management.

This study has certain limitations that also present opportunities for future research. First, the number of USV species may be underestimated because the survey was conducted during a single season. Conducting surveys across multiple seasons could help detect species that emerge or establish at different times of the year. Second, the discussion on the potential roles of USV, such as pollinator support (Figure 4) and soil stabilization (Figure 5), is based on field observations made during the plant surveys. Future studies employing systematic assessments are needed to better understand these roles and to explore additional ecosystem functions provided by USVs. Third, this study provides qualitative data in the form of a species list without information on species abundance. Future studies focusing on species abundance and the environmental factors influencing the abundance and distribution of USVs would give deeper insights into effective management in urban ecosystems.

## 4. Materials and Methods

### 4.1. Study Area

The study focused on the rapidly developing city of Chiang Mai. Chiang Mai is in northern Thailand along the Ping River (18.78° N latitude and 98.98° E longitude). The city has an average elevation of 313 m above sea level [119]. In 2023, Chiang Mai received approximately 1188 mm of rainfall [120], with temperatures ranging from 22 °C to 34 °C [121]. As one of the fastest-growing cities, Chiang Mai has seen significant expansion in urban communities, industrial sectors, and the tourism industry, driving both economic and social development in the region [122,123,124].

**Figure 6:**
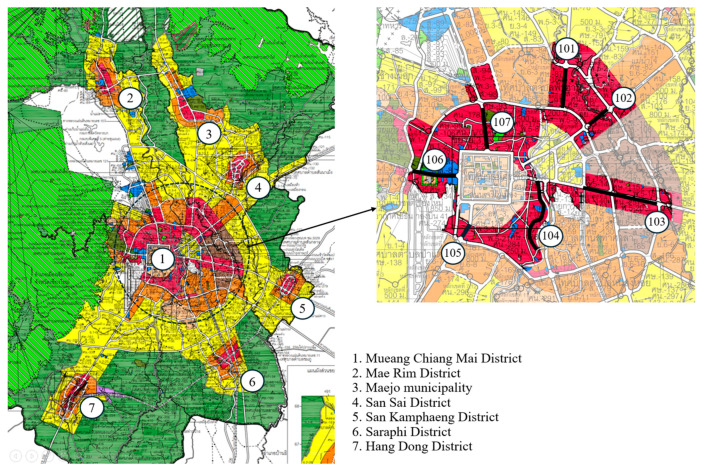
Seven survey locations in urban high-density residential and commercial zones (**left**) and seven surveyed sidewalk routes (bold black lines, numbered 101–107) within Location (1) Mueang Chiang Mai District (**right**).

The studied areas were busy roads in urban high-density residential and commercial zones (red colored zones, Figure 6). The surveyed areas were classified as high-density residential and commercial zones by the Ministry of Interior B.E. 2564 (2021). There were seven sampling locations: (1) Mueang Chiang Mai District (23.12 km), (2) Mae Rim District (2 km), (3) Maejo municipality (1.56 km), (4) San Sai District (0.76 km), (5) San Kamphaeng District (2.56 km), (6) Saraphi District (0.92 km), and (7) Hang Dong District (2.32 km) (Figure 6). The total linear distance was 33.24 km.

### 4.2. Data Collection and Analysis

To determine species composition and characteristics of USV on urban sidewalks, a sampling zone (a sidewalk) was defined as a linear space bounded on one side by a road. The survey covered 13 sidewalk routes. Each location consisted of a single survey route, which was a main road with heavy traffic, except for the location in Mueang Chiang Mai District, which had seven surveyed routes (Figure 6). The plant survey was conducted from June to July 2023, a rainy season in Thailand.

We walked along the sidewalk, and the species of plants growing within the sidewalk area were recorded. For all species found in the sampling zones, plant parts were collected for voucher specimens and identified using the taxonomic literature (i.e., *Flora of Thailand*, *Flora of China*, *Flora of Java*, and *Thai Forest Bulletin*). The growth habits of USVs were divided into lying flat, upright, and climbing. Other growth habits not included in this division were recorded separately [125,126,127,128] (Table 2). The life cycles were divided into annual and perennial. The growth habits and life cycles of USVs were counted and compared. In addition, we identified non-native and native species based on Plants of the World Online, facilitated by the Royal Botanic Gardens, Kew [129]. Alien invasive species were identified using the list of alien invasive plants in Thailand [130].

## 5. Conclusions

This study provides the first comprehensive survey of urban spontaneous vegetation (USV) in Chiang Mai, Thailand, focusing on their species composition and potential use in urban greening. A total of 63 USV species from 24 families were identified, with Poaceae and Asteraceae as the dominant families. The widespread occurrence of certain species across all surveyed sites demonstrates their adaptability to harsh urban conditions, while the predominance of annual species highlights their capacity to colonize disturbed and compacted soils rapidly.

Although this study did not directly assess ecological functions or ecosystem services, field observations indicate that some USV species contribute to urban biodiversity and provide habitats for animals. Many species are drought-tolerant and have low-maintenance traits, suggesting their suitability for enhancing urban green spaces, particularly in fragmented areas where trees cannot establish, such as small urban gardens, roadside verges, and median strips.

More than half of the USV species observed were alien species, some of which may threaten native biodiversity by outcompeting local species and disrupting ecological interactions such as pollination and seed dispersal. These insights highlight the need for targeted management to minimize the ecological risks posed by invasive alien species while harnessing the functional and aesthetic benefits of USV. Future research should focus on developing long-term strategies for integrating USV into urban landscapes, including the identification of resilient and beneficial species, the assessment of pavement maintenance practices, and the implementation of adaptive regimes across varied urban contexts.

## Figures and Tables

**Table 1:** USV species recorded in this survey, their native status, their growth habit and life cycle, and some of their pharmacological activities (NAs in the pharmacological activities and references column indicate no pharmacological activities have been reported).

No.	Species	Family	Native Status	Growth Habit	Life Cycle	Some Pharmacological Activities	References
1	*Acalypha indica* L.	Euphorbiaceae	Native	Erect	Annual	Anthelmintic, anti-ulcer, antibacterial, asthma, bronchitis, and wound healing	[28]
2	*Acalypha lanceolata* Willd.	Euphorbiaceae	Native	Erect	Annual	NA	NA
3	*Alternanthera pungens* Kunth	Amaranthaceae	Alien	Prostrate	Perennial	Anti-inflammatory and antioxidant	[29]
4	*Alternanthera sessilis* (L.) DC.	Amaranthaceae	Native	Decumbent	Perennial	Anti-asthmatic, anti-hypertensive, and anti-spasmodic	[30]
5	*Amaranthus viridis* L.	Amaranthaceae	Invasive	Erect	Annual	Antiallergic, antihepatotoxic, anti-inflammatory, antiulcer, and antiviral	[31]
6	*Axonopus compressus* (Sw.) Beauv.	Poaceae	Invasive	Stoloniferous	Perennial	Antibacterial, antifungal, and antioxidant	[32]
7	*Bidens pilosa* L.	Asteraceae	Invasive	Erect	Annual	Anti-allergy, anti-hypertensive, anti-inflammatory, anti-malarial, anti-microbial, and antioxidant	[33]
8	*Boerhavia diandra* L.	Nyctaginaceae	Alien	Prostrate	Annual	NA	NA
9	*Boerhavia diffusa* L.	Nyctaginaceae	Native	Decumbent	Annual	Anticancer, antidiabetic, anti-inflammation, antifibrinolytic, diuresis, and immunomodulation	[34]
10	*Boerhavia repens* L.	Nyctaginaceae	Native	Prostrate	Annual	Anticonvulsant, antifungal, anti-inflammatory, antiulcer, and anxiolytic	[35]
11	*Bothriochloa ischaemum* (L.) Keng	Poaceae	Native	Geniculate	Perennial	NA	NA
12	*Cenchrus brownii* Roem. & Schult.	Poaceae	Alien	Decumbent	Annual	NA	NA
13	*Cenchrus echinatus* L.	Poaceae	Alien	Geniculate	Annual	Anti-inflammatory and antiproliferative	[36]
14	*Chloris barbata* Sw.	Poaceae	Native	Stoloniferous	Perennial	Antibacterial, anti-diabetic, and antimicrobial	[37]
15	*Chromolaena odorata* (L.) R.M.King & H.Rob.	Asteraceae	Invasive	scandent	Perennial	Antibacterial, anti-inflammatory, antifungal, antioxidant, and cytotoxic	[38]
16	*Cleome rutidosperma* DC.	Cleomaceae	Invasive	Procumbent	Annual	Anti-inflammatory, anti-microbial, antioxidant, diuretic, laxative, and wound healing	[39]
17	*Coccinia grandis* (L.) Voigt	Cucurbitaceae	Native	Climbing	Perennial	Anti-inflammatory, antimicrobial, antioxidant, hepatoprotective, hypoglycemic, and mutagenic	[40]
18	*Commelina benghalensis* L.	Commelinaceae	Native	Decumbent	Annual	Anti-inflammatory, anti-urolithiasis, antimicrobial, antioxidant, antiviral, and hepato-protective,	[41]
19	*Cyanthillium cinereum* (L.) H.Rob.	Asteraceae	Native	Erect	Annual	Anti-inflammatory, antimicrobial, and antioxidant	[42]
20	*Cynodon dactylon* (L.) Pers.	Poaceae	Native	Stoloniferous	Perennial	Anti-inflammatory, antimicrobial, antiparasitic, antioxidant, antiviral, and wound healing	[43]
21	*Cynodon nlemfuensis* Vanderyst	Poaceae	Alien	Stoloniferous	Perennial	NA	NA
22	*Cyperus compressus* L.	Cyperaceae	Native	Tussock	Annual	Antidiabetic, antidiarrheal, antimalarial, antimicrobial, antioxidant, and hypotensive	[44]
23	*Cyperus rotundus* Linn.	Cyperaceae	Native	Tussock	Perennial	Analgesic, antibacterial, anticancer, antidiabetic, anti-inflammatory, antioxidant, and weight control	[45]
24	*Dactyloctenium aegyptium* (L.) Willd.	Poaceae	Native	Stoloniferous	Annual	Anticancer, anti-inflammatory, antioxidant, antipyretic properties, and gastrointestinal effects	[46]
25	*Eclipta prostrata* (L.) L.	Asteraceae	Alien	Erect	Annual	Antibacterial and antioxidant	[47]
26	*Eleusine indica* (L.) Gaertn.	Poaceae	Native	Geniculate	Annual	Antibacterial, antifungal, anti-inflammatory, antioxidant, antiviral, and hepatoprotective	[48]
27	*Emilia sonchifolia* (L.) DC.	Asteraceae	Native	Ascending	Annual	Anti-inflammatory, anti-ulcer, antioxidant, immunomodulatory, and wound healing	[49]
28	*Eragrostis tenella* (L.) P.Beauv. ex Roem. & Schult.	Poaceae	Native	Geniculate	Annual	NA	NA
29	*Erigeron floribundus* (Kunth) Sch. Bip.	Asteraceae	Invasive	Erect	Annual	Anti-inflammatory and immunomodulatory	[50]
30	*Euphorbia bifida* (Hook. & Arn.)	Euphorbiaceae	Native	Erect	Annual	NA	NA
31	*Euphorbia hirta* L.	Euphorbiaceae	Invasive	Erect or prostrate	Annual	Antibacterial, anti-inflammatory, antifungal, antioxidant, and wound healing	[51,52]
32	*Euphorbia thymifolia* L.	Euphorbiaceae	Alien	Prostrate	Perennial	Antibacterial, antifungal, anti-inflammatory, antimicrobial, antioxidant, and larvicidal	[53]
33	*Evolvulus nummularius* (L.) L.	Convolvulaceae	Invasive	Prostrate	Perennial	Antibacterial, anticonvulsant, antihelminthics, antioxidant, and wound healing	[54]
34	*Gomphrena celosioides* Mart.	Amaranthaceae	Invasive	Prostrate	Perennial	Antiarthritic and antihyperalgesic	[55]
35	*Grona triflora* (L.) H.Ohashi & K.Ohashi	Fabaceae	Native	Prostrate	Perennial	Antiproliferative and antioxidant	[56]
36	*Indigofera hendecaphylla* Jacq.	Fabaceae	Native	Prostrate	Perennial	NA	NA
37	*Ipomoea cairica* (L.) Sweet	Convolvulaceae	Native	Climbing	Perennial	Anti-inflammatory, antioxidant, antiviral, and highly potent against malaria	[57]
38	*Ipomoea obscura* (L.) Ker Gawl.	Convolvulaceae	Native	Climbing	Perennial	Anti-inflammatory, antibacterial, and anti-tumor	[58,59]
39	*Leptopetalum pteritum* (Blume) Neupane & N.Wikstr.	Rubiaceae	Native	Ascending	Annual	NA	NA
40	*Malvastrum coromandelianum* (L.) Garcke	Malvaceae	Alien	Erect	Annual	Analgesic, antibacterial, anti-inflammatory, and antinociceptive	[60]
41	*Melochia corchorifolia* L.	Malvaceae	Native	Decumbent	Annual	Anticancer, antibacterial, antioxidant, antiurolithiatic, CNS stimulant, and diuretic	[61]
42	*Oldenlandia corymbosa* L.	Rubiaceae	Native	Erect or procumbent	Annual	Abortifacient effects, antioxidant, cytotoxic, hepatoprotective, and antimicrobial	[62]
43	*Oxalis corniculata* L.	Oxalidaceae	Invasive	Ascending	Perennial	Anticancer, antidiabetic, antinociceptive, hepatoprotective, and hypolipidemic	[63]
44	*Passiflora foetida* L.	Passifloraceae	Invasive	Climbing	Annual	Antibacterial	[64]
45	*Phyllanthus amarus* Schumach. & Thonn.	Phyllanthaceae	Invasive	Erect	Annual	Anticancer, anti-inflammatory, antimicrobial, antiplasmodial, antibacterial, antioxidant, antiviral, and nephroprotective	[65]
46	*Physalis angulata* L.	Solanaceae	Alien	Erect	Annual	Anticancer, antidiabetic, anti-inflammatory, antifibrotic, antibacterial, and antiparasitic	[66]
47	*Pilea microphylla* (L.) Liebm.	Urticaceae	Alien	Ascending	Annual	Antibacterial and antioxidant	[67,68]
48	*Portulaca oleracea* L.	Portulacaceae	Alien	Prostrate	Annual	Anti-fertility, antiulcerogenic, antimicrobial, antioxidant, and bronchodilator	[69]
49	*Portulaca pilosa* L.	Portulacaceae	Alien	Prostrate or ascending	Annual	Analgesic, anti-inflammatory, anti-ulcerogenic, antibacterial, antioxidant, and wound healing	[70]
50	*Rivina humilis* L.	Petiveriaceae	Invasive	Erect	Annual	Antimicrobial and antioxidant	[71]
51	*Ruellia prostrata* Poir.	Acanthaceae	Alien	Procumbent	Perennial	Antibacterial, anti-inflammatory, antioxidant	[72]
52	*Ruellia tuberosa* L.	Acanthaceae	Invasive	Erect	Perennial	Anti-inflammatory, antifungal, hypoglycemic, hypolipidemic, antimicrobial, and wound healing	[73]
53	*Setaria flavida* (Retz.) Veldkamp	Poaceae	Native	Geniculate	Perennial	Antioxidant	[74]
54	*Spermacoce remota* Lam.	Rubiaceae	Invasive	Erect	Perennial	NA	NA
55	*Sporobolus diandrus* (Retz.) P.Beauv.	Poaceae	Native	Tussock	Perennial	NA	NA
56	*Synedrella nodiflora* (L.) Gaertn.	Asteraceae	Invasive	Erect	Annual	Analgesic, anti-inflammatory, antimicrobial, antioxidant, and antipyretic	[75]
57	*Talinum fruticosum* (L.) Juss.	Talinaceae	Invasive	Erect	Annual	Anti-bacterial, anti-inflammatory, anti-tumor, anticarcinogenic, antioxidant, and antiviral	[76]
58	*Tribulus terrestris* L.	Zygophyllaceae	Native	Prostrate	Annual	Anti-inflammatory, anti-tumor, anti-urolithic, antidiabetic, and antioxidant	[77]
59	*Tridax procumbens* L.	Asteraceae	Invasive	Procumbent	Perennial	Anti-inflammatory, anti-tumor, anti-urolithic, antidiabetic, and antioxidant	[78]
60	*Turnera ulmifolia* L.	Turneraceae	Invasive	Erect	Perennial	Anti-inflammatory and antimicrobial	[79,80]
61	*Urochloa distachyos* (L.) T.Q.Nguyen	Poaceae	Native	Stoloniferous	Perennial	Anthelmintic	[81]
62	CF-USV7 *	Poaceae	Unknown	Stoloniferous	Unknown	Unknown	Unknown
63	CF-USV9 *	Poaceae	Unknown	Erect	Unknown	Unknown	Unknown

* CF-USV7 and CF-USV9 were unidentified species.

**Table 2:** The category and characteristics of plant growth habits.

Growth Habit Category	Characteristics	Example of Growth Habits
Lying flat	Stems grow flat on the ground. Some may root at nodes to spread across a large area. Tend to form ground cover.	Procumbent, decumbent, prostrate, stoloniferous, rhizomatous, rosette.
Upright	Stems grow straight up from the base. Do not need external structures to remain upright.	Ascending, erect, tussock, scandent, virgate, intricate, divaricate, suckers, coppice shoots.
Climbing	Do not grow upright on their own but instead use support structures. Often use tendrils, aerial roots, or twining stems to climb. Can grow to great heights and cover large areas.	
Other	Irregular growth.	Geniculate.

## Data Availability

Data are contained within the article.
